# The Influence of Chemical Chaperones on Enzymatic Activity under Thermal and Chemical Stresses: Common Features and Variation among Diverse Chemical Families

**DOI:** 10.1371/journal.pone.0088541

**Published:** 2014-02-10

**Authors:** Michal Levy-Sakin, Or Berger, Nir Feibish, Noa Sharon, Lee Schnaider, Guy Shmul, Yaniv Amir, Ludmila Buzhansky, Ehud Gazit

**Affiliations:** Department of Molecular Microbiology and Biotechnology, Tel Aviv University, Tel Aviv, Israel; Instituto Tecnologia Quimica e Biologica; Universidade Nova de Lisboa, Portugal

## Abstract

Molecular and chemical chaperones are key components of the two main mechanisms that ensure structural stability and activity under environmental stresses. Yet, chemical chaperones are often regarded only as osmolytes and their role beyond osmotic regulation is not fully understood. Here, we systematically studied a large group of chemical chaperones, representatives of diverse chemical families, for their protective influence under either thermal or chemical stresses. Consistent with previous studies, we observed that in spite of the structural similarity between sugars and sugar alcohols, they have an apparent difference in their protective potential. Our results support the notion that the protective activity is mediated by the solvent and the presence of water is essential. In the current work we revealed that i) polyols and sugars have a completely different profile of protective activity toward trifluoroethanol and thermal stress; ii) minor changes in solvent composition that do not affect enzyme activity, yet have a great effect on the ability of osmolytes to act as protectants and iii) increasing the number of active groups of carbohydrates makes them better protectants while increasing the number of active groups of methylamines does not, as revealed by attempts to synthesize *de novo* designed methylamines with multiple functional groups.

## Introduction

The function of organisms under stress is a fundamental physiological task. Proteins are especially sensitive to misfolding and denaturation induced by extreme conditions including high or low temperatures, high hydrostatic pressure, high ionic strength and the presence of chemical denaturants such as urea. Environments characterized by those states may significantly alter protein structure and thus result in loss of function. There are two known biological mechanisms of adaptation that enable cell survival under such harsh environments. It is well known that when exposed to stress, cells up-regulate molecular chaperones levels. Molecular chaperones are widely known for their ability to interact with and stabilize misfolded proteins and polypeptides, but also as key players in protein transport to specific organelles, protein degradation, protein disaggregation and more [1], [2]. Nevertheless, an alternative less–explored strategy to avoid protein denaturation exists in cells and organisms from all kingdoms of life, ranging from simple bacteria to human. This strategy includes the accumulation of small naturally occurring organic molecules, known as osmolytes [3]–[5]. Osmolytes affect cell osmosis and play a pivotal role in the maintenance of cell volume, oxidative state, redox balance and energy storage. Osmolytes can be found at very high concentrations: The intracellular levels of trimethylamine *N*–oxide (TMAO) can reach 300 mM in deep sea animals [6]. Even higher concentrations can be found in the Australian desert mouse in which the kidney cells can accumulate up to 5 M of urea and 2.5 M of counteracting osmolytes [7], [8]. Many studies over the last few decades indicated that besides osmoregulation, these compounds also have an important role in the stabilization of proteins [9]–[11]. Thus, the term ‘chemical chaperones’ is often used to describe the diverse collection of osmolytes serving as protecting agents [12]. Although first described already in the early 1970s [13], the exact mechanism by which chemical chaperones induce stabilization of protein structure is still under debate [5], [14]–[19]. An additional approach for the stabilization of proteins is by the addition of their native ligands, cofactors or agonists (also termed pharmacological chaperones) which can stabilize proteins by specific binding [20], [21]. They can stabilize mutant proteins or even native proteins that are susceptible to unfolding/misfolding under physiological conditions, and even induce folding of unfolded proteins [22], [23].

There is a large variation among chemical chaperones, both in structure and origin. They are commonly classified by their chemical composition into four major groups: Methylamines, amino acids, sugars and polyols [24], [25]. While distinctively different from one another, all these substances were found to accumulate in cells living in extreme surroundings, or in response to different kinds of stresses. It is not known whether the same adaptation mechanism evolved several times in parallel, or specific groups of molecules were selected through the evolutionary forces for defined stresses. Despite variations in their chemical properties, it was suggested that a universal physicochemical mechanism explains their ability to stabilize proteins. Transfer free energy measurements have demonstrated that osmolytes stabilizing effects stem mainly from their influence on protein backbone and only marginally from their impact on protein side chains [26]–[29]. Thus, they affect mainly the unfolded state, in which the backbone is more exposed. Protecting osmolytes interact unfavorably with the unfolded state while urea, a destabilizer interacts favorably with the protein backbone. These insights were utilized by Auton and Bolen who further developed Tanford's transfer model for calculating and predicting cooperative folding and unfolding free energy changes in the presence of osmolytes [27], [28], [30].

Although believed that the effect of chemical chaperones is universal, and that a protective effect can be observed even for proteins that are not naturally found in the presence of organic solutes [31], there is accumulating evidence that methylamines for instance, are better suited for the perturbation of proteins by urea [32], while polyols are better used as cryoprotectants and thermal stabilizers [33]. Even in common laboratory usage enzymes are often stored in liquid formulations of polyols (mainly glycerol) in order to maintain their function.

Various model proteins were used to study stabilization by chemical chaperones in terms of both structure and function. Proteolytic enzymes are often chosen as models for such works since it is easy to track and measure their activity. Different studies have shown the positive effects of various chemical chaperones on the structure and activity of lysozyme [4], [10], [34], chemotrypsin [35], [36] and trypsin [36]. In the latter, Thompson and co–workers assessed whether the chemical chaperone TMAO may serve as a better agent to preserve trypsin and indeed, they found that the tryptic activity was enhanced in the presence of TMAO [36]. Trypsin is a digestive enzyme which cleaves peptides and proteins exclusively at the carboxyl side of lysine or arginine residues [37]. The activity of trypsin can be readily detected by the use of the N_α_–Benzoyl–D,L–arginine 4–nitroanilide (BAPNA) synthetic substrate. Hydrolysis of BAPNA releases the *p*–nitroaniline chromophore which can be followed by spectroscopic analysis [38].

In the current work, trypsin was used to screen the ability of different chemical chaperones to preserve enzymatic activity under different stress conditions. The results revealed that polyols are better stabilizers for trypsin than sugars in the case of heat stress, while sugars were found to counteract much more efficiently the trifluoroethanol (TFE) chemical denaturant. Furthermore, the length of the carbon chain, or the number of hydroxyl groups, both in polyols and in sugars, were found to have a major role in the efficiency of the compounds as chemical chaperones. On the other hand, synthesis of *de novo* designed methylamines with additional functional groups diminished their protective effect. Moreover, the presence of water was found to be essential for the chemical chaperones to be effective.

## Materials and Methods

Adonitol, betaine anhydrous, chlorocholine chloride, choline chloride, D–maltose monohydrate, D–arabinose, D–arabitol, D–glucose, D–mannitol, D–mannose, D–sorbitol, D–threitol, D–xylose, diethylene glycol, ectoine, ethyleneglycol, glycine, L–alanine, L–arabinose, L–arabitol, L–arginine, L–lysine, L–proline, maltose, myo–inositol, sarcosine, taurine, TMAO, xylitol and β–alanine were purchased from Sigma–Aldrich. D–galactose was purchased from Frutarum. D–raffinose and trehalose were purchased from Acros. Glycerol was purchased from Gadot.

### Circular dichroism analysis

Far–UV circular dichroism (CD) spectra were obtained using an Applied Photophysics Circular Dichroism Spectrometer equipped with a temperature–controlled sample holder. Spectra were collected between 180 nm and 250 nm at 1 nm intervals, with averaging time of 4 seconds per point. Only data with optical density lower than 2 were used. Data is expressed as mean residue molar ellipticity [θ]: [θ]  =  θ×100×M/(n×C×l), where θ is the observed ellipticity, M is the molecular mass, n is number of amino acids, C is the concentration in mg/ml, and l is the path length in cm. [θ] is given is deg×cm^2^×dmol^-1^

Trypsin CD measurements were carried out in 20 mM Potassium phosphate buffer, 150 mM NaCl pH 8.3 at a concentration of 0.06 mg/ml using 2 mm quart cuvette (Hellma, Germany). Samples were allowed to equilibrate for 5 minutes at each temperature before measurements.

### Trypsin heat denaturation

Each chemical chaperone was dissolved in 20 mM Tris–HCl (pH 8.3); 150 mM NaCl to its maximal concentration and then six dilutions were prepared (87.5%, 75%, 50%, 37.5%, 25% and 12.5% v/v). Trypsin from bovine pancreas (Sigma–Aldrich) was dissolved freshly before each experiment in 20 mM Tris–HCl (pH 8.3); 150 mM NaCl to a concentration of 3 mg/ml and added to each solution to a final concentration of 125 µg/ml, including a control with no chemical chaperone. In order to prevent autodigestion of trypsin all samples were kept on ice. One ml of each solution was heated to 60°C for 10 minutes. 120 µl of each heated sample were transferred to a 96–wells plate for an enzymatic activity assay.

### Trypsin chemical denaturation

Each chemical chaperone was dissolved in 30% TFE, 70% 20 mM Tris–HCl (pH 8.3); 150 mM NaCl solution to its maximal concentration, and then six dilutions were prepared (87.5%, 75%, 50%, 37.5%, 25% and 12.5% v/v) using a 30% TFE, 70% 20 mM Tris–HCl (pH 8.3); 150 mM NaCl solution. Trypsin was added to each solution to a final concentration of 125 µg/ml, including a control with no chemical chaperone. 120 µl of each treated sample were transferred to a 96–wells plate for an enzymatic activity assay.

### Trypsin enzymatic activity assay

15 mg of the chromogenic substrate, BAPNA (Nα–Benzoyl–D,L–arginine 4–nitroanilide HCl) (Fluka) was dissolved in 500 µl of DMSO, and then diluted in 59.5 ml of 20 mM Tris–HCl (pH 8.3); 150 mM NaCl to a final concentration of 0.3 mg/ml. Immediately before the activity measurements 130 µl of the substrate was added to the 96–wells plate (containing triplicates of each sample). The kinetic readings consisted of measuring absorbance at 414 nm in a Synergy HT plate reader (Bio–Tek) every 30 seconds for 5 minutes at 25°C. The slope of the produced curve was calculated in order to quantify the enzymatic activity of trypsin. The activity of an untreated enzyme was determined as the native activity. The activity of denatured enzyme with no chemical chaperones was determined as baseline and was subtracted from the other measures. All measures were calculated as percentages of the native activity.

### *De novo* designed chemical chaperones synthesis

Synthesis of *N,N,N′,N′*–Tetramethylpropylenediamine *N*–oxide (CC1) and *N*,*N*,*N′*,*N′*–tetramethyl–ethane–1,2–diamine *N*–oxide (CC2) was carried out by oxidation of *N,N,N′,N′*–Tetramethyl–1,3–propanediamine (TMPDA) and tetramethylethylenediamine (TEMED) respectively according to Cope and Ciganek [39] with a few modifications. Briefly, 0.35 mole of TMPDA or TEMED (Sigma–Aldrich), 0.7 mole of hydrogen peroxide (Sigma–Aldrich) and 45 ml of methanol (Bio–Lab Ltd.) were placed in a flask. The solution was allowed to stand at room temperature for 36 hours. Portions of 0.7 mole hydrogen peroxide were added after 2 and 4 hours. Formation of the product was confirmed by NMR and mass spectrometry.

## Results and Discussion

### Secondary structure of trypsin

To study the protective effect of chemical chaperones on the trypsin model enzyme, we selected two different types of denaturation – thermal and chemical, both leading to loss of activity.

**Figure 1:**
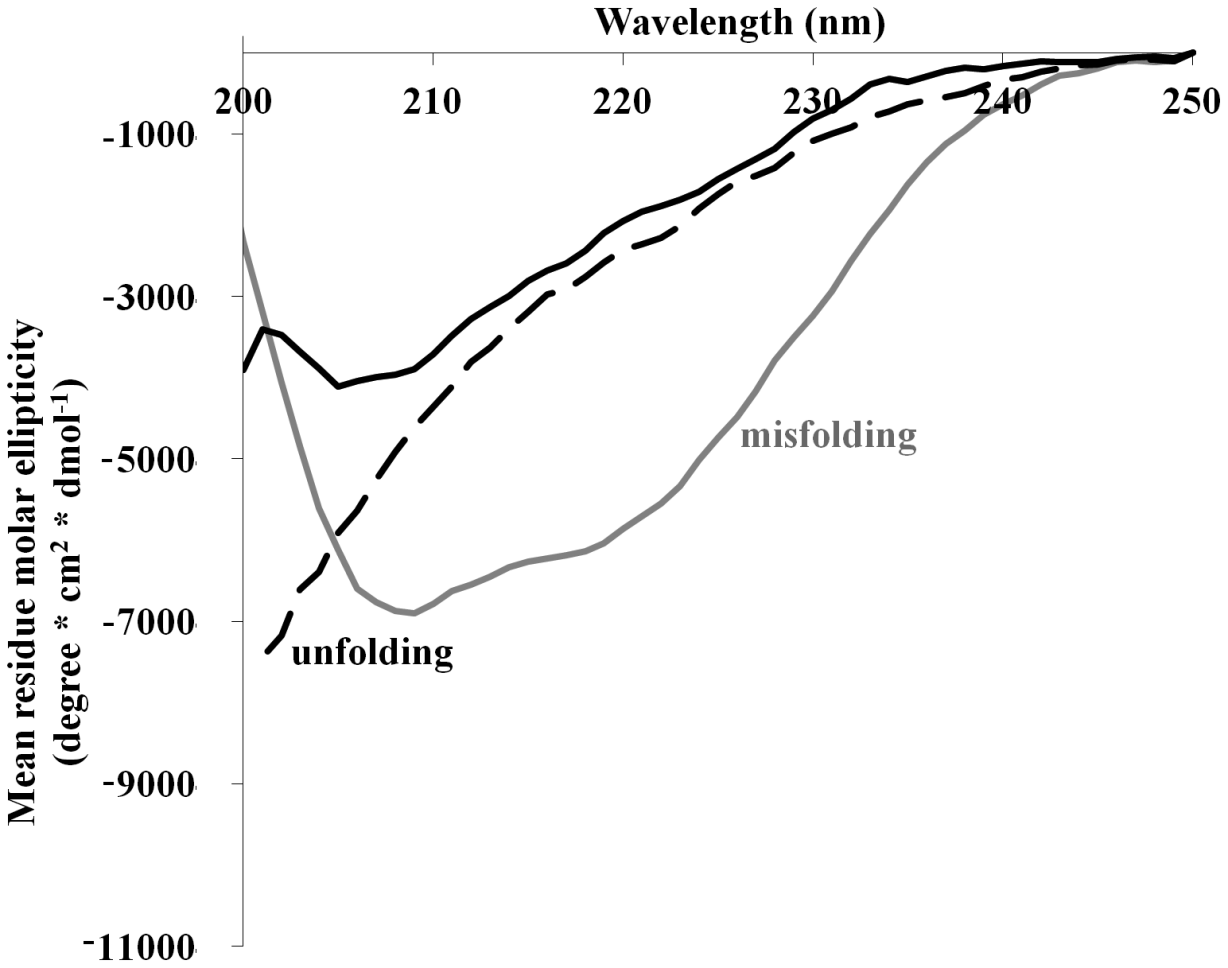
Trypsin structure under denaturating conditions. 0.06/ml trypsin was dissolved in buffer alone (solid black line) and in the presence of 30% TFE (gray line) or at 60°C (dashed line). In the presence of TFE, the CD spectrum showed misfolding while under heating an unfolded state was observed.

Trypsin is easily denatured by heating to 60°C for 10 minutes, resulting in an unfolded state as indicated by circular dichroism (CD) spectroscopy. Trypsin remains unfolded even after cooling down back to room temperature (Figure 1) and shows a complete loss of activity (assay will be further described). Since trypsin does not refold upon cooling, thermodynamic analysis, including m-values calculations, according to the transfer energy model could not be applied in this study.

Another way to readily denature proteins is with the use of common chemical denaturants such as urea and guanidine HCl (GuHCl) [3], [40]. However, these denaturants could not be used in this study. Trypsin was treated with 1–8 M urea and complete inactivation was observed only at 7–8 M. The high concentrations of urea that are required to inactivate trypsin did not allow the preparation of urea and chemical chaperones co–solutions which would be sufficient for returning trypsin activity. On the other hand, GuHCl is a specific inhibitor of the enzyme and could not be used in an enzymatic assay: Even when as low as 100 mM GuHCl was used only 5% of trypsin activity was restored (using 4 M TMAO). TFE which is known to promote structural changes in proteins, mainly as a highly potent helix inducer [41], was chosen instead. Complete loss of enzymatic activity of trypsin was achieved at 30% TFE and as expected, an increase in the amount of α–helical structures was indicated by CD measurements (Figure 1). This allowed the screening of the ability of chemical chaperones to protect trypsin against two kinds of different states: Unfolding as the result of thermal stress and misfolding induced by TFE.

### Stabilization of trypsin with chemical chaperones under heat denaturation

**Figure 2:**
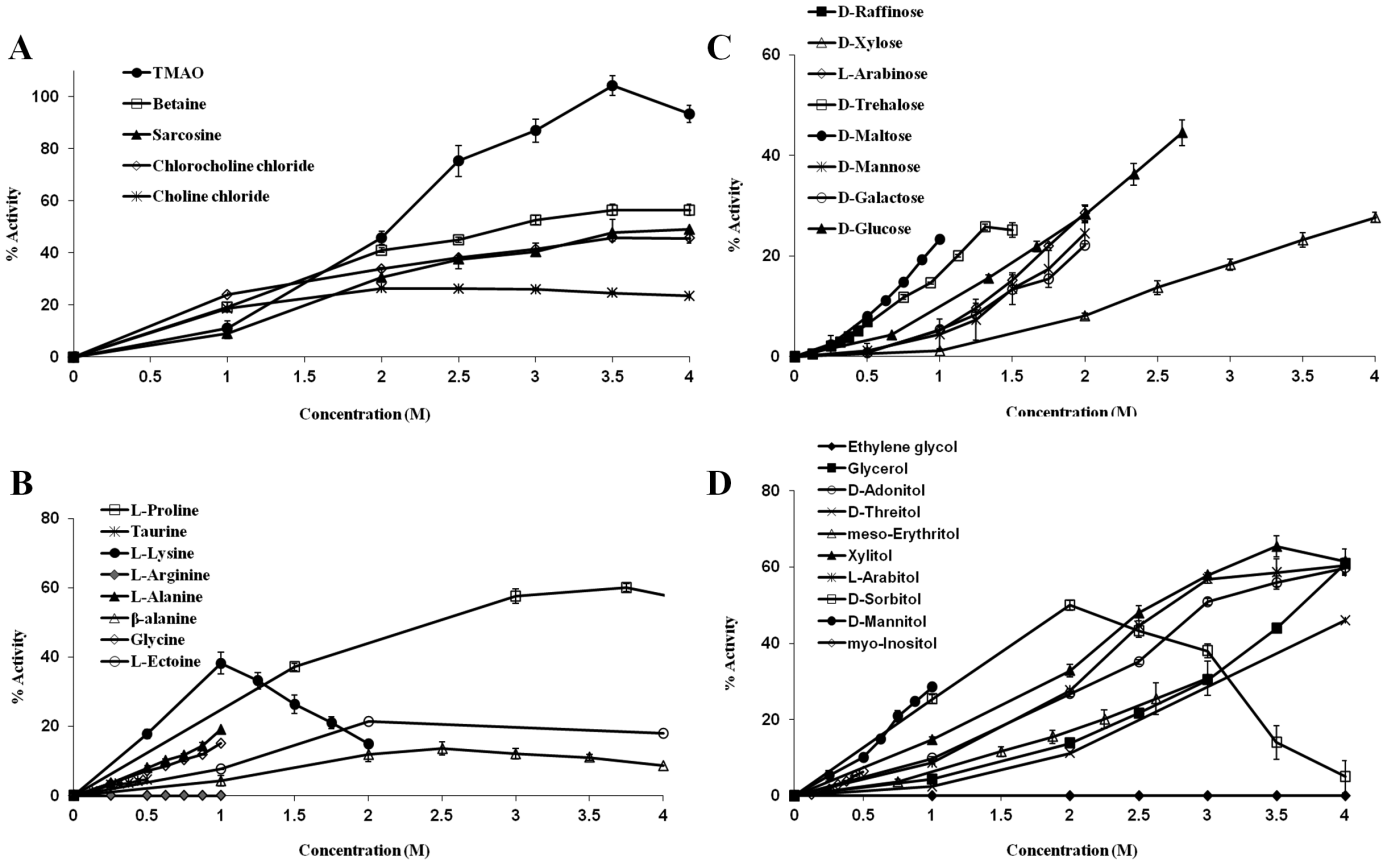
Activity of heated trypsin following incubation with chemical chaperones of different chemical families. The activity of heated trypsin following incubation with: (A) methylamines, (B) amino acids, (C) sugars, (D) polyols. 125 µg/ml trypsin was denaturated and inactivated by a 10 minutes heating to 60°C alone or in the presence of chemical chaperones. After adding BAPNA as a substrate absorbance at 414 nm was read every 30 seconds for five minutes and trypsin activity was derived from the slope of the measurement. 100% activity represents non-heated trypsin.

We measured the activity of trypsin after heat treatment in the presence of chemical chaperones at different concentrations (up to 4 M). Briefly, 125 µg/ml trypsin was incubated for 10 minutes at 60°C in the absence or presence of chemical chaperones. After the enzyme solution was cooled to reach room temperature, BAPNA was added and enzymatic activity was measured. Over 30 different osmolytes were tested and the results were classified according to the main known groups of chemical chaperones: Methylamines, amino acids, sugars and polyols (Figure 2).

#### Methylamines

Of all the compounds tested, TMAO was significantly the most effective, able to fully preserve the activity of trypsin at 3.5 M. Other chemical chaperones from the methylamines group were only able to preserve about 50% of the native activity of trypsin (betaine 56%; sarcosine 49% and chlorocholine chloride 46%) or less (chlorine chloride 26%). A plateau was reached for some of the methylamines, starting from 3.5 M for betaine and chlorocholine chloride and 2 M for choline chloride (Figure 2A). Although in natural systems, methylamines are found in association with high levels of urea and other inorganic ions or in correlation with increased hydrostatic pressure [42], their ability to increase the thermostability of proteins is a well–established observation [10], [43]–[45]. Our results are in agreement with previous studies.

#### Amino acids

Amino acids and their derivatives are the dominant solutes in salt–tolerant bacteria, marine invertebrates and many plants [25], [46]. The L–stereoisomers of the amino acids were used in this assay since they are more commonly found in cells as both osmolytes and building blocks. Due to relatively low solubility of some amino acids, lower concentrations were examined compared to other compounds (Figure 2B). L–proline was the most efficient amino acid in protecting trypsin under the heat treatment, able to preserve a maximum of 60% of its native activity at 3.75 M. At the lower range of concentrations of up to 1 M, L–lysine seemed to be the most potent out of the amino acids group, preserving over 35% of the enzymatic activity. Yet, at higher concentrations a decrease in activity was observed. As mentioned above, trypsin cleaves proteins exclusively at the carboxyl side of lysine or arginine. This specific side–chain recognition of trypsin is mediated by interaction of the negatively charged aspartate 189, located at the substrate binding pocket, with the positively charged lysine or arginine [47]. It is possible that at high concentrations, free lysine is also recognized by the enzyme and acts as a competitive inhibitor to BAPNA, and thus a decrease in BAPNA hydrolysis was noticed. Further examination of our data supports this notion, since lysine induced a decrease in the activity of trypsin even when the enzyme was not denaturated (data not shown). Similar results were also obtained with L–arginine, the other moiety recognized by trypsin. L–arginine was not able to preserve trypsin's activity after heating, and it drastically decreased its activity even without heating (data not shown). A few natural non–coded amino acids were examined as well, including taurine, a solute occurring at high levels in marine invertebrates and mammalian neurons [24], [48]; β–alanine, an osmolyte that was found to have a protective effect on cells during hypoxia [24] and is also known to suppress heat inactivation of proteins [49]; and ectoine, a cyclic amino acid accumulated in bacteria in response to heat or salt stress [50]. These three amino acids were less efficient than most of the coded amino acids examined in this assay, preserving 20% and less of the native activity of trypsin. Amino acids that were previously shown to increase the thermal stability of proteins include: Glycine, L–proline, L–serine, taurine, α–alanine, β–alanine and L–arginine [10], [51], [52]

#### Carbohydrates

**Figure 3:**
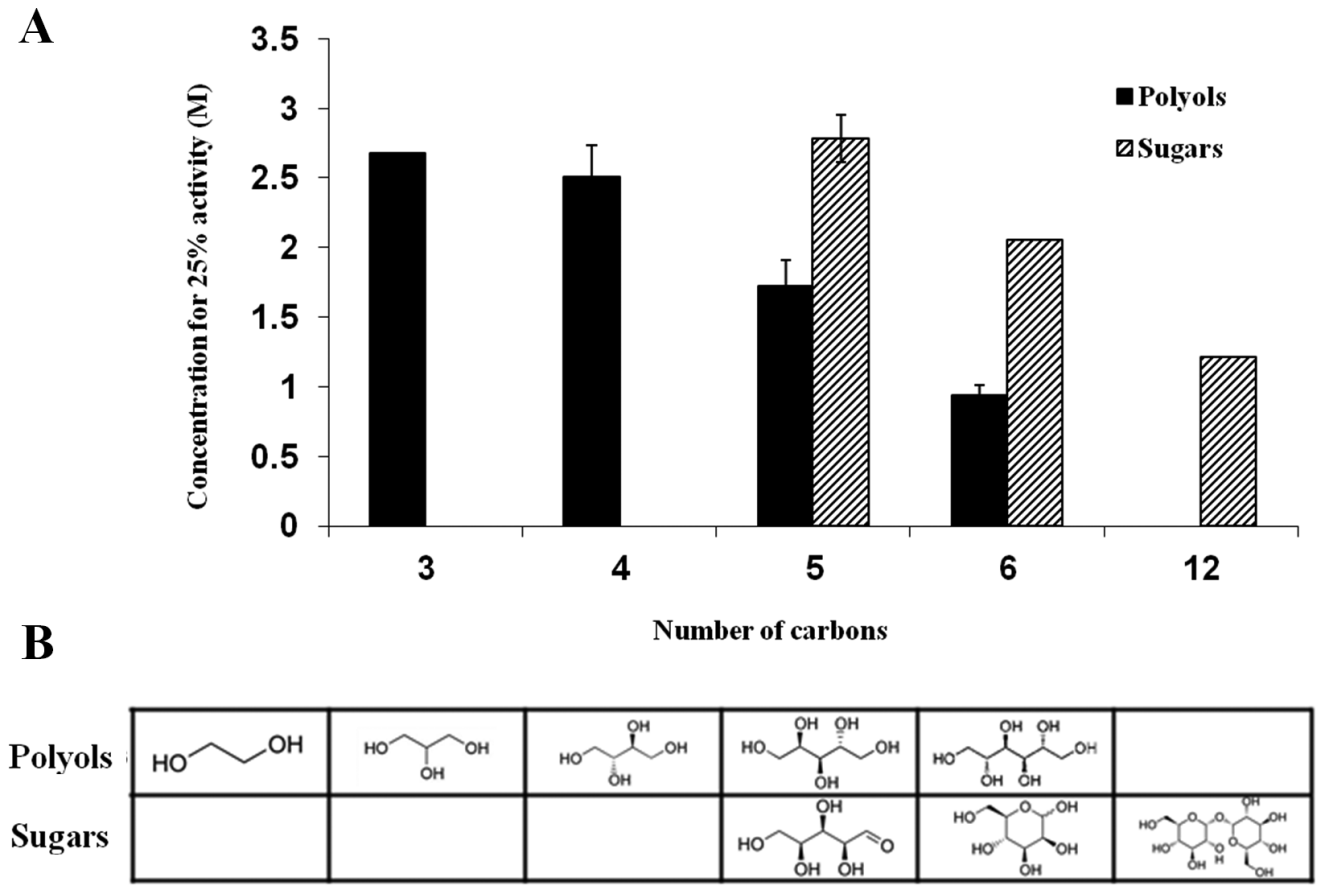
Comparison between the ability of sugars and polyols to protect trypsin from activity loss following heating. (A) The concentration of various polyols and sugars that is required for restoring 25% activity of heated trypsin is presented as a function of the compounds carbon chain lengthy. (B) Examples for polyols and sugars with varying carbon chain lengths.

Sugars and polyols (also known as sugar alcohols) are small carbohydrates that comprise most of the accumulating molecules in freeze–avoiding organisms [24]. Both sugars and polyols were found to preserve the enzymatic activity of trypsin under the heat treatment (Figure 2C–D). Most of the carbohydrates acted in a dose–dependent manner, with the exception of sorbitol which was the most potent of the group at 2 M but became less efficient as its concentration was raised. Since the polyol and sugar molecules are very similar, and only differ from one another by the number of carbons in their backbone and the number of hydroxyl groups presented, we decided to examine the association between these attributes and the ability of the molecules to stabilize trypsin's activity under stress. Nine polyols of different backbone lengths were examined in this assay: Two carbons (ethylene glycol), three carbons (glycerol), four carbons (D–theritol, *meso*–erythritol), five carbons (D–adonitol, L–arabitol, xylitol) and six carbons long (D–sorbitol, D–mannitol). We compared the concentration of the polyol needed to preserve 25% of the native activity, a set point achieved by all of the examined carbohydrates, except for ethylene glycol which could not induce 25% activity of trypsin after heating at any concentration (Figure 3). While other polyols are known as protein stabilizers ethylene glycol was shown as protein stabilizer in some cases [53] and as a destabilizer in others [33], [54], indicating that a threshold for the length of a polyol to act as a protectant may exist. A similar comparison was made for the various examined sugars containing: Five carbons (D–xylose, L–arabinose), six carbons (D–glucose, D–galactose, D–mannose) and twelve carbons (D–trehalose, D–maltose). Two main trends can be indicated by this analysis: a) the ability of polyols and sugars to suppress heat inactivation of trypsin is positively influenced by the molecule size; b) polyols are better stabilizers than sugars in preventing heat denaturation of trypsin. Our findings are in clear agreement with previous studies in which polyols and sugars increased the T_m_ of variety of proteins and retained their enzymatic activity under non–physiological conditions. Correlation between the number of hydroxyl groups and the stabilizing effect was demonstrated for both polyols [55]–[57] and sugars [18], [55].

The influence of polyols and sugars on protein stability was argued in the past to be the outcome of their influence on surface tension [56], [58], water structure [55], water activity, steric exclusion, solvophobicity and hydrophobicity [33], as reviewed by Kumar et al. [59]. It was suggested that the free energy at the protein water interface increase due to higher tension levels in the presence of polyols leading to polyols exclusion from the protein. However, glycerol stabilizes proteins while it lowers the water surface tension [56], [60]. Lately it was postulated that the hydrophobicity of the polyol/sugar rather than the simply the number of hydroxyl groups accounts for their stabilizing effect [33]. However, the correlation between hydrophobicity and the influence on protein folding was demonstrated separately for polyols and sugars, indicating that hydroxyl content is not sufficient to predict the protective effect of carbohydrates on protein structure and function.

#### Secondary structure

**Figure 4:**
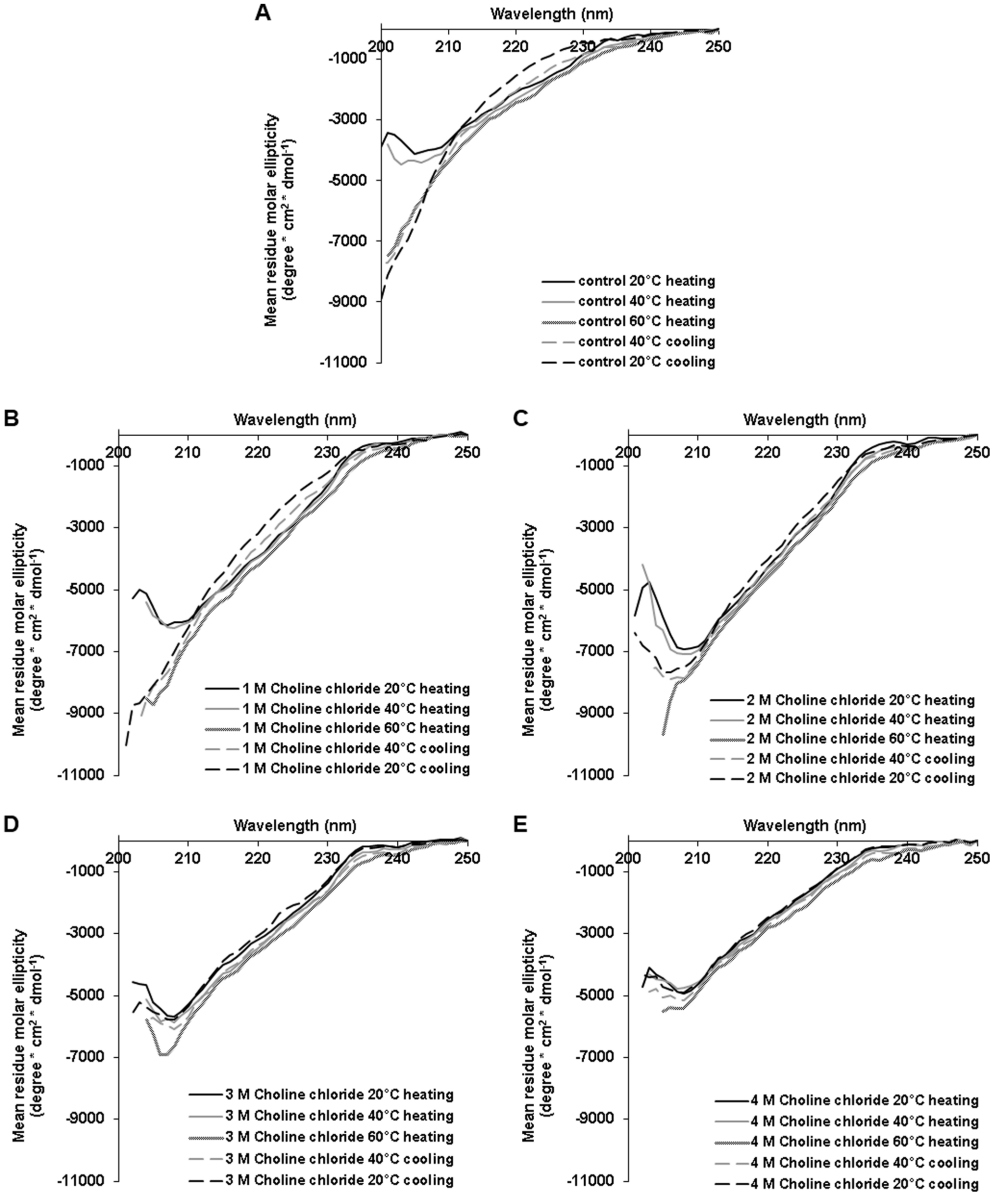
Trypsin secondary structure in the process of heat denaturation in the presence and absence of choline chloride. 0.0625/ml trypsin was heated from 20°C to 60°C in (A) buffer solution alone or in the presence of choline chloride (B) 1 M, (C) 2 M, (D) 3 M and (E) 4 M and cooled down back to 20°C. CD spectra were taken at 20°C, 40°C and 60°C during the heating process and again at 20°C and 40°C during the cooling process.

Next, we used CD spectroscopy to detect trypsin secondary structure changes during heat denaturation in the presence of chemical chaperones. Trypsin was mixed with the methylamine choline chloride at various concentrations and CD spectra were taken at 20°C, 40°C, 60°C and again after the sample was cooled down to 40°C and to 20°C. We monitored trypsin structure in the presence of rising concentrations of choline chloride (0–4 M). At the presence of 1 M choline chloride, trypsin unfolding was irreversible (Figure 4B), similar to the control sample (buffer without chemical chaperones, Figure 4A). At 2 M choline chloride partial refolding was detected (Figure 4C) and at higher concentrations (3 M and 4 M) only minor unfolding was observed. Thus, after cooling back to 20°C trypsin was well folded (Figure 4C–D). It is worth mentioning that there is no correlation between the observed secondary structure and the relative activity (Figure 2A). According to the later assay choline chloride restored trypsin activity but reached a plateau from 1 M onwards (of about 25% activity). Such a trend is not reflected from the CD data. These measurements, in combination with enzymatic activity data provide information regarding the denaturation transition states of trypsin and their activities. It can be stated that in the presence of choline chloride, different denaturation transition states give rise to similar enzymatic activity (2 M – 26.35±0.33%, 3 M – 25.95±0.47%, 4 M – 23.4±0.62%).

More often studies on protein stabilization in the presence of osmolytes focus on protein folding rather than protein activity. Here, we demonstrated conditions in which full recovery was observed in terms of structure but not function. This is probably due to small structural changes that could not be measured by CD. Our results emphasize the importance of monitoring enzymatic activity in addition to analysis of the folding state of the protein.

**Figure 5:**
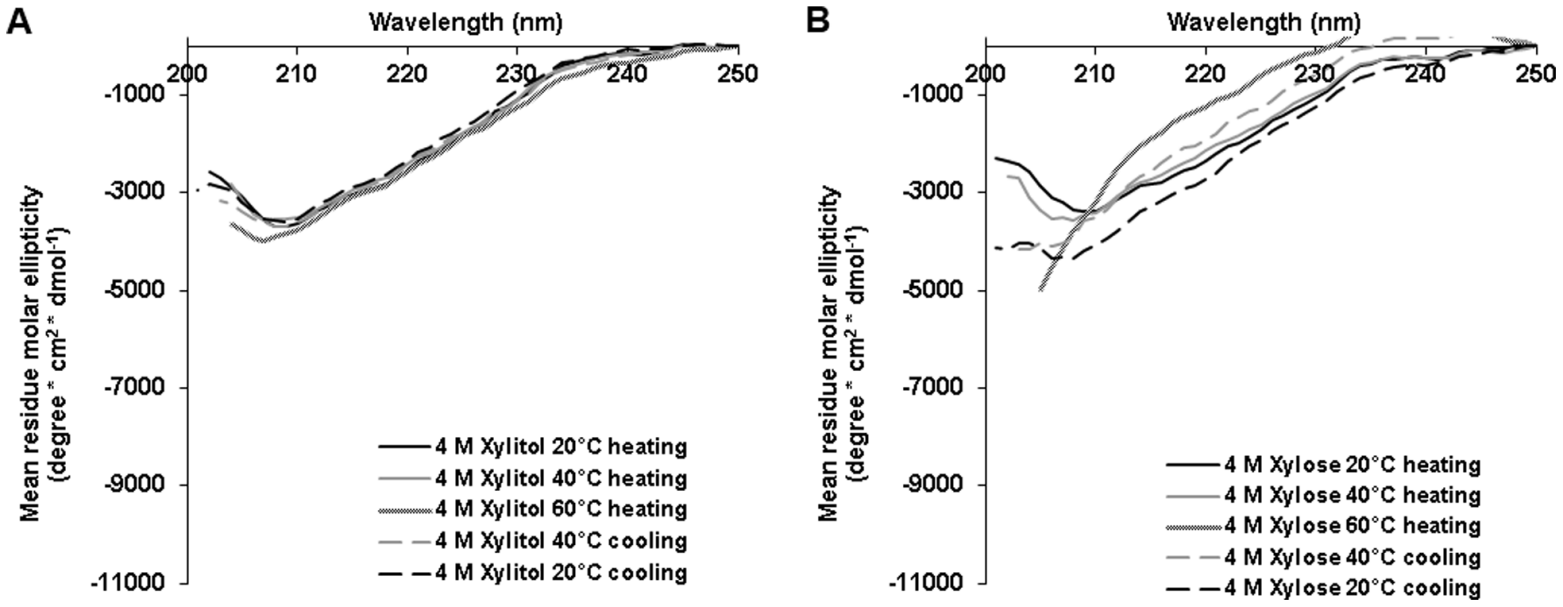
Trypsin secondary structure during heat denaturation in the presence of xylitol and xylose. 0.06/ml trypsin was heated from 20°C to 60°C in the presence of 4 M (A) xylitol and (B) xylose and cooled down back to 20°C. CD spectra were taken at 20°C, 40°C and 60°C during the heating process and again at 20°C and 40°C during the cooling process.

Next, we monitored the influence of the sugar xylose and the polyol xylitol on trypsin structure, both at 4 M (Figure 5). As was demonstrated by activity assay, xylose was less efficient than xylitol in preserving trypsin activity upon heating. Here, CD measurements were in agreement with this finding. When trypsin was heated to 60°C in the presence of 4 M xylose the protein was partially unfolded. After cooling back to 20°C partial refolding of the protein was observed (Figure 5B). In the presence of 4 M xylitol, trypsin had a more folded structure with only minor changes in its secondary structure at 60°C. After cooling back to 20°C its CD spectrum was highly similar to the spectrum that was monitored before heating, indicating that the changes in conformation were largely reversible (Figure 5A). Due to high optical activity at the range of the relevant wavelength (200–250 nm), none of the amino acids was chosen for CD measurements.

### Stabilization of trypsin with chemical chaperones under chemical denaturation

**Figure 6:**
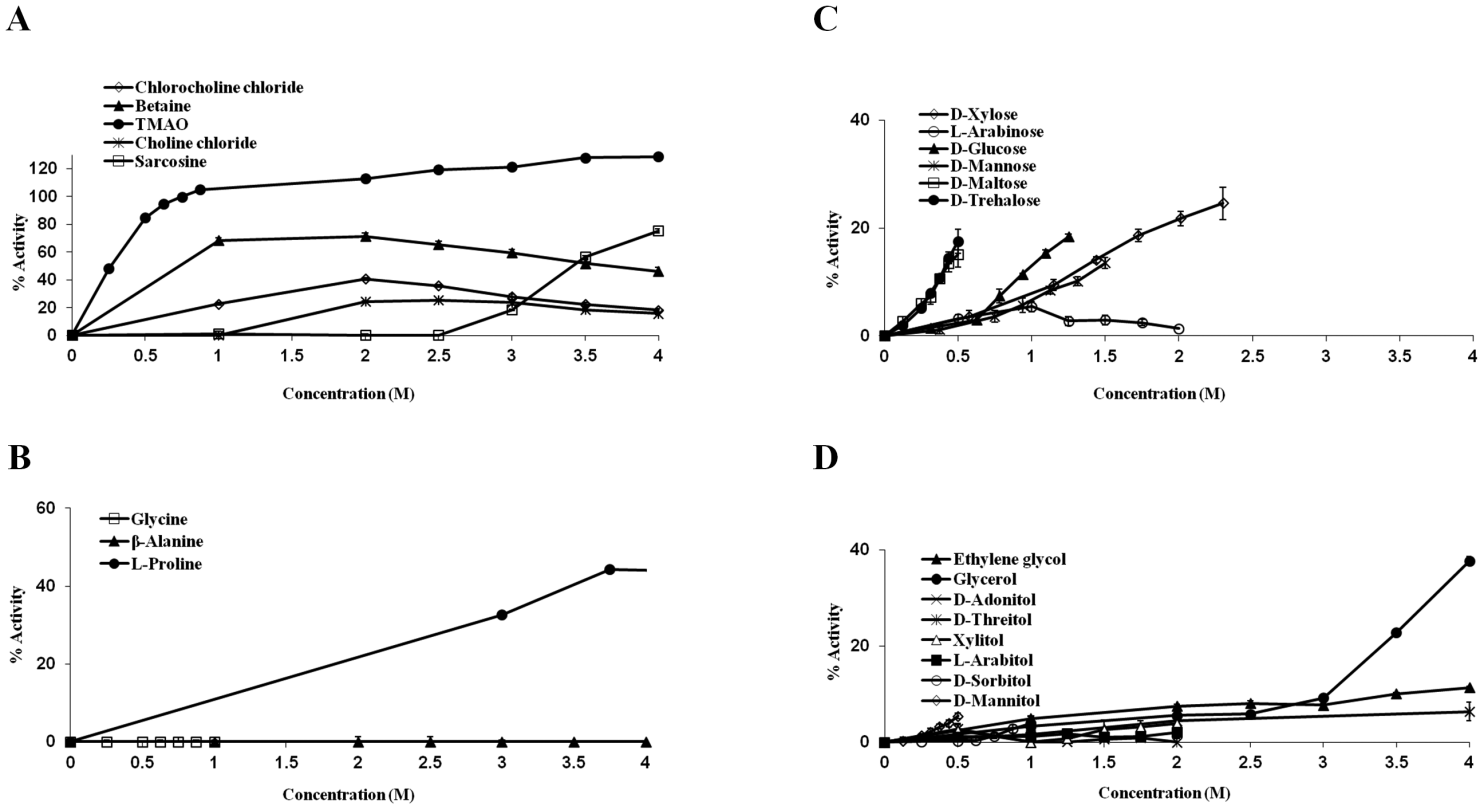
Activity of trypsin under chemical stress following incubation with chemical chaperones of different chemical families. The activity of trypsin under chemical stress, using 30% TFE when incubated with: (A) methylamines, (B) amino acids, (C) sugars and (D) polyols. Trypsin, 125 µg/ml was inactivated by the addition of 30% TFE. Trypsin was incubated in the presence or in the absence of chemical chaperones. After adding BAPNA as a substrate, absorbance signal at 414 nm was recorded every 30 seconds for five minutes and trypsin activity was derived from the slope of the measurements. 100% activity represents non-heated trypsin.

The screening of the ability of chemical chaperones to protect trypsin against chemical denaturation was performed by the incubation of the enzyme in buffer solution containing 30% TFE in the absence or presence of the different chemical chaperones, followed by an enzymatic activity assay (Figure 6).

Similar to the heat denaturation experiments, the most efficient compound to protect trypsin in this assay was once again TMAO (Figure 6A). The presence of TMAO led to full recovery of the enzymatic activity, even at concentrations lower than 1 M. This result is in agreement with the known role of TMAO and other methylamines as counteracting agents to the effect of urea and other denaturants [24]. The methylamines in general were the best protectants against chemical denaturation by TFE among the four examined groups.

Due to low solubility in the presence of TFE, only three amino acids were tested. Glycine and β–alanine had no detectable protective effect on trypsin inactivation at 30% TFE. On the contrary, L–proline was efficient in counteracting the effect of TFE, and in its presence up to 45% of the activity was preserved (Figure 6B).

Sugars do not dissolve well at 30% TFE, thus, our measurements were limited to low sugar concentrations. However, regardless of the lack of solubility, the tested sugars were able to restore up to 20% activity of trypsin. In contrast, polyols, which were shown to be more efficient agents than sugars to counteract heat stress, and are more soluble, were almost completely inefficient in neutralizing the chemical stress (Figure 6C–D).

**Figure 7:**
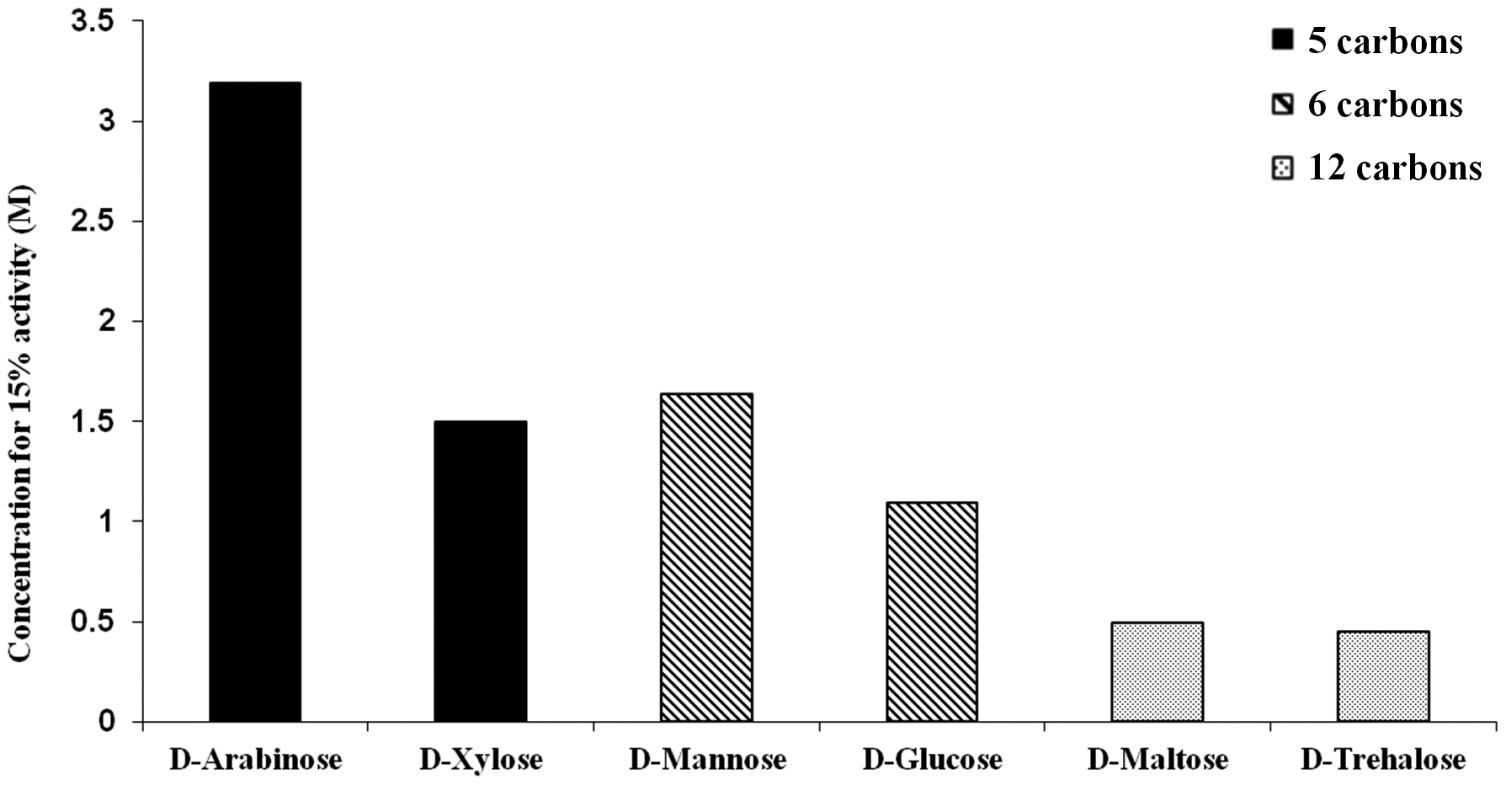
Comparison of the ability of different sugars to protect trypsin's activity, due to chemical denaturation induced by the presence of 30% TFE. The compounds are compared by the required concentration for restoring 15% activity of trypsin.

In the case of heat denaturation a positive influence of the size of the carbohydrate molecules on their ability to stabilize trypsin was observed. When the stabilizing effect was studied in the context of chemical stress, a similar trend was observed for sugars alone. Longer chained sugars were more efficient at counteracting TFE. This is demonstrated by the sugar concentration required in order to gain 15% of the native enzymatic activity of trypsin, a set point achieved by all of the examined sugars (Figure 7). In the case of polyols, merely ethylene glycol was able to reach this set point, the only polyol to have no effect on trypsin under heat stress.

In order to understand the distinct effect of polyols and sugars on the tryptic activity in the presence of TFE, one should consider the complex network of interactions: TFE–water, TFE–protein, osmolyte–water, osmolyte–protein and osmolyte–TFE. Our system provides only a hint on the result of these interactions and higher resolution methods or molecular dynamic simulation studies may shed light on further details. Based on previous studies, TFE was found to influence proteins mainly through two mechanisms: a. Formation of TFE clusters leading to local hydrophobic areas which induce destabilization of protein hydrophobic interactions; and b. Formation of hydrogen bonds with the carbonyl group of the protein backbone which cause protein destabilization [61]. Here, we demonstrated that sugars are better counteracts of TFE than polyols. It is possible that TFE interacts with the carbonyl moiety of the sugars, competing with the TFE–backbone interactions and diminishing the fraction of TFE molecules available to form hydrophobic patches around the protein. Furthermore, the presence of a halogen group in TFE enables it to form stronger hydrogen bonds than polyols, making the polyol molecules weak competitors on water and protein interactions [61].

### *De novo* designed chemical chaperones

**Figure 8:**
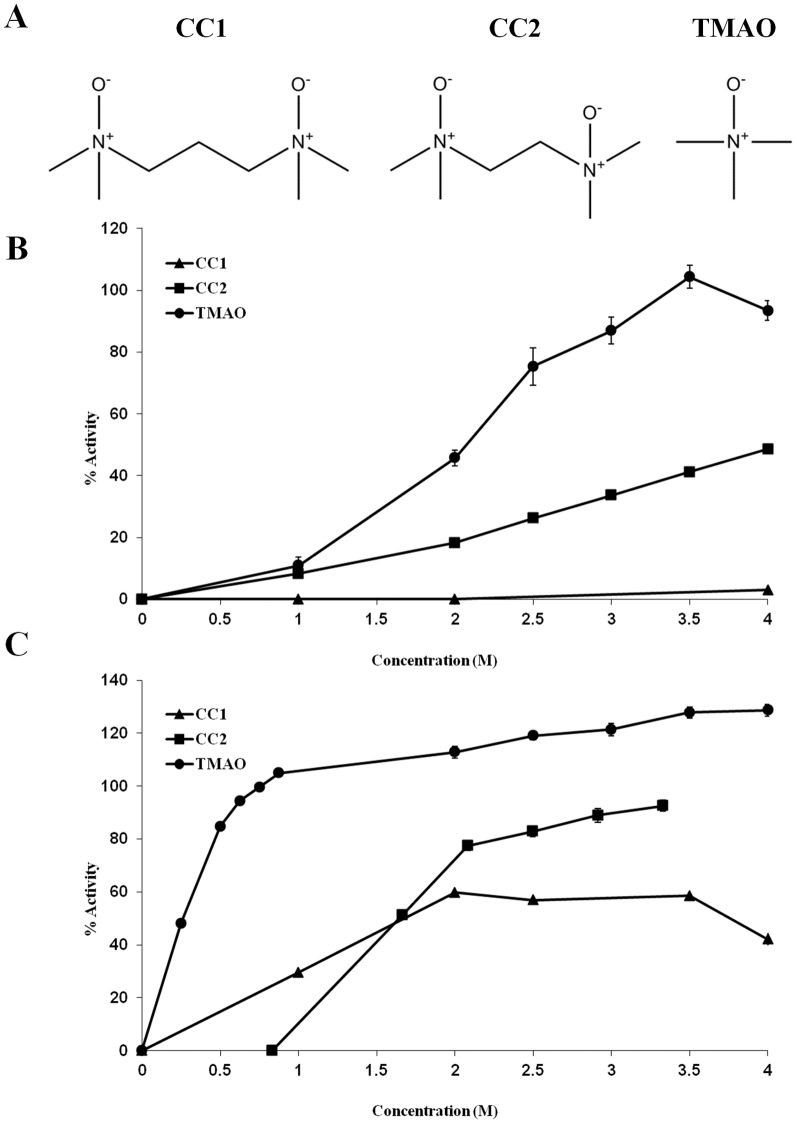
*De novo* designed chemical chaperones. Two new chemical chaperones were designed and synthesized, corresponding to (A) two attached TMAO molecules, with a carbon linker, CC1, or without a linker, CC2. The potency of the compounds was assessed using trypsin (B) heat denaturation assay and (C) chemical denaturation assay.

Based on the screenings results, we designed new compounds that may act as improved chemical chaperones. We demonstrated for polyols and sugars that an increase in the number of hydroxyl groups improves the potency of the carbohydrate as a stabilizer that counteracts heat stress. Similar results were also obtained when sugars were used to negate the effect of chemical stress. As for methylamines, to the best of our knowledge there are no naturally occurring osmolytes bearing more than one functional group. Therefore, we designed two new compounds: *N*,*N*,*N′*,*N′*–Tetramethylpropylenediamine *N*–oxide and *N*,*N*,*N′*,*N′*–Tetramethyl–ethane–1,2–diamine *N*–oxide, termed CC1 and CC2, respectively (Figure 8A). The structures of the two *de novo* designed compounds are equivalent to two TMAO molecules fused together, CC1 with one carbon as a linker and CC2 without a linker. CC1 and CC2 were synthesized, purified by flash chromatography (the degree of purity was confirmed by analytical HPLC) and characterized by mass spectrometry and ^1^H NMR.

To evaluate the efficiency of CC1 and CC2 as chemical chaperones, we used trypsin heat and chemical denaturation assays (as previously described). Solutions of 0–4 M CC1, CC2 and TMAO were used (Figure 8B–C). While TMAO could fully recover the activity of heat treated trypsin, CC1 did not protect the activity at all and CC2 recovered about 50% activity. When chemical denaturation assay was applied, all the three methylamines: TMAO, CC1 and CC2 protected trypsin from activity loss. Again, TMAO completely recovered trypsin's activity, CC1 was the least efficient one, with maximal recovery of 60% activity, and CC2 exhibited almost full recovery of trypsin's activity (92% activity). Thus, although CC1 and CC2 acted as chemical chaperones, the introduction of a second functional group to TMAO did not improve its efficiency. Our results also indicate that the linker length between the functional groups is important for the activity of the compound as a chemical chaperone.

A new high resolution model for TMAO-water interactions by Larini and Shea [62] revealed novel dynamical and structural properties of this system, which may shed light on our results. They demonstrated that the methyl groups of neighboring TMAO molecules repeal each other. Such repulsion is not possible in the case of the CC1 and CC2 since here, two methyl groups are physically attached. In contrary to the repulsion of the methyl groups, TMAO attracted two to three water molecules. The surrounding water molecules were repulsed together with their accompanying methyl groups. It was suggested that this special network of attraction and repulsion forces leads to the formation of a unique water structure around TMAO molecules which results in the exclusion of TMAO molecules from the protein surface. In light of these findings, it would be interesting to further investigate the influence of the linker between the methyl groups on the protective activity of such compounds.

### Chirality of chemical chaperones

**Figure 9:**
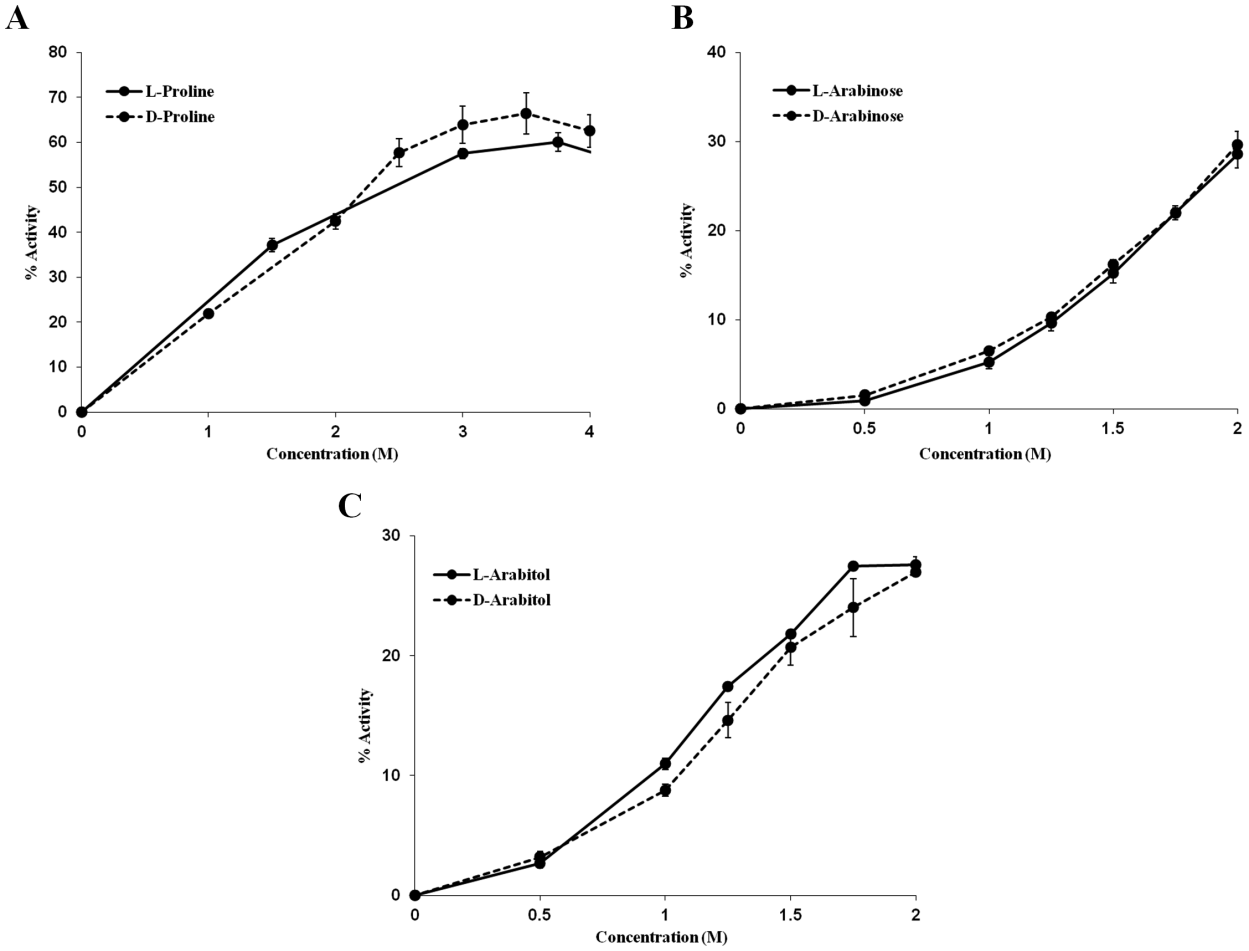
Chirality of chemical chaperones. Stereo-isomers of chemical chaperones stabilize trypsin equivalently, as demonstrated by trypsin heat denaturation in the presence of (A) L-proline and D-proline, (B) L-arabinose and D-arabinose, or by (C) trypsin chemical denaturation in the presence of L-arabitol and D-arabitol.

A key assumption regarding chemical chaperones is that they stabilize proteins and other macromolecules through non–specific interactions. Thus, the chirality of an osmolyte should not affect its ability to protect proteins from denaturation. On the contrary, as one would expect, when specific interactions are involved, the chirality of a molecule interacting with a protein is crucial. Therefore, we compared the ability of stereo–isomers to stabilize trypsin. L–proline and D–proline (Figure 9A) as well as L–arabinose and D–arabinose (Figure 9B) were shown to be able to protect trypsin from heat denaturation in the same manner. L–arabitol and D–arabitol were shown to protect trypsin from chemical denaturation in a similar manner (Figure 9C). Thus, the three examples that were studied support the notion that chemical chaperones interact in a non–stereospecific manner with trypsin. These results, as well as additional observations previously obtained in other studies, further support the notion that chemical chaperones interactions are not direct and thus are mediated by the solvent, mainly water [18]. For further understanding of the influence of the solvent, we were interested to study whether we could alter the protective effect of chemical chaperones by modifying the solvent.

### Solvent effects on chemical chaperones

**Figure 10:**
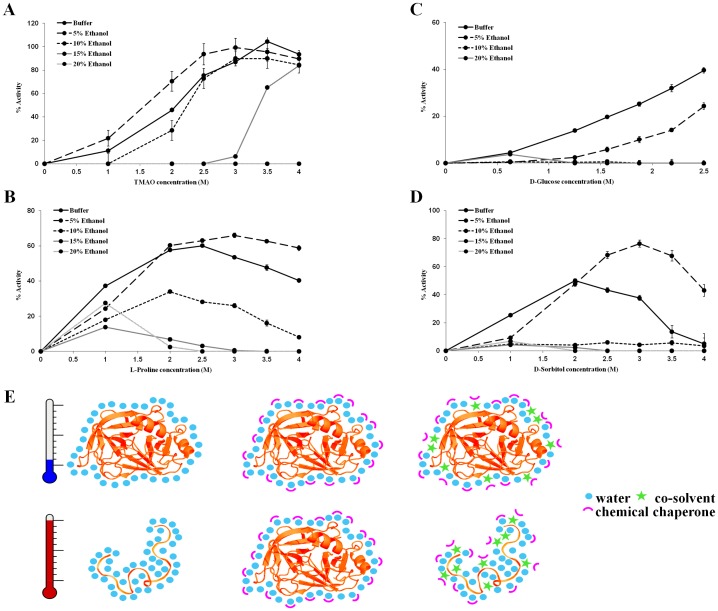
The influence of ethanol as a co-solvent on chemical chaperones' ability to protect trypsin against heat denaturation. Trypsin activity was measured following heating to 60°C in the presence of 0%–20% ethanol with (A) TMAO, (B) L-proline, (C) D-glucose and (D) D-sorbitol. (E) A model for the role of co-solvents in the activity of chemical chaperons. Preferential exclusion of chemical chaperons stabilizes the folded state by increasing the hydration shell of the protein. The co-solvent interferes with the integrity of the hydration shell surrounding the protein and prevents the preservation effect of the chemical chaperons upon heating.

When dissolved in ethanol (up to 70%), the activity of trypsin remains the same. Moreover, heating trypsin in the presence of ethanol (up to 20%) gives similar results to those acquired when heating trypsin in buffer (data not shown). Hence, the activity of trypsin is not affected by ethanol in these conditions. Therefore, ethanol can be used as a co–solvent to monitor solvent effects on the protective potential of chemical chaperones. The notion that the stabilizing properties of an osmolyte are dependent on the solvent composition was addressed in the past, but mainly in terms of solvent pH [63], [64]. To this end, heat denaturation experiments were performed as described above, with the exception of the presence of ethanol (up to 20%) in the buffer (Figure 10).

When incubated with 0–4 M TMAO and 20% ethanol followed by heating, trypsin became completely inactive. At 15% ethanol, enzymatic activity was observed only at TMAO concentrations above 3.5 M. When the percentage of ethanol was 10% or less, the efficiency of TMAO followed a similar pattern to the experiment with no co–solvent, while a slight improvement was observed at 5% ethanol. Similar negative effect on the efficiency of the chemical chaperone was revealed when examining representatives from the other chemical groups; the amino acid L–proline, the sugar D–glucose and the polyol D–sorbitol (Figure 10B–D). In general, ethanol as a co–solvent acted as a suppressor of the activity of chemical chaperones (with the exception of 5% ethanol which had a partially improving effect).

Solvent effect studies of ethanol and other small monohydric alcohols reveal a complex impact of alcohol content on protein structure and function: a. At low concentrations ethanol can lead to the formation of a compact and more stable protein conformation, due to increase in the hydrophobic effect, b. In similar to TFE, ethanol can induce the formation of non–native α–helical structures and c. At high concentrations, ethanol can induce unfolding of proteins [65], [66]. The contradictory observation that at low ethanol concentration (5%) trypsin activity after heating can be higher than the measured activity in buffer conditions may be the result of the stabilizing effect of ethanol at low concentrations, as described above [65]. Vibrational spectroscopy studies of lysozyme in water–ethanol mixed solutions revealed that at low ethanol concentrations (0.15–0.18 ethanol molar fraction) changes in protein–solvent interactions can be detected but they do not lead to protein conformational modifications [67], [68]. Taken together with the profound effect of ethanol on the ability of the studied osmolytes to act as protective agent, it is likely that by altering the hydrogen bond network of the protein with the surrounding water, ethanol also altered the ability of the osmolytes to act as chemical chaperones. Molecular dynamics simulations demonstrated that monohydric alcohols tend to accumulate mainly in the vicinity of the protein, occupying major part of the first hydration shell and form hydrogen bonds directly with the protein [69]. In the same study polyhydric alcohols were shown to be excluded from the protein and thus remain mainly in the bulk water. Solutes stabilizing effect is often attributed to their preferential exclusion from the protein, leading to a more hydrated protein with a more compact and stable conformation of the macromolecule [17], [29], [69]–[71]. The presence of monohydric alcohols at the first hydration shell of the protein may attenuate the ability of the chemical chaperones to promote a hydrated and compact conformation of the protein. This is in agreement with our data and with other accumulating evidence for the non–direct effect of chemical chaperones on proteins which appears to be mediated by proteins' natural solvent–water (for a tentative model see Figure 10E).

To conclude, we provide new insights on the mechanism of chemical chaperone activity under stress conditions, especially on the role of functional groups and solvent effect. This is another step towards better understanding of the evolvement and function of this ubiquitous yet diverse system. Our unsuccessful attempts to design improved chemical chaperones further illustrate the optimization of the current method as achieved by the process of evolution under ever–changing conditions. There is still an open question regarding the need and development of such a wide range of agents, sequentially or in parallel.
